# Current German Recommendations and International Research on the Use of COVID-19 Boosters among Health Care Providers in 2024: A Narrative Review

**DOI:** 10.3390/medicina60030385

**Published:** 2024-02-25

**Authors:** Poramate Pitak-Arnnop, Popchai Ngamskulrungroj, Nithi Mahanonda, Prim Auychai, Benjamin Frech, Veronika Shavlokhova, Christian Stoll

**Affiliations:** 1 Department of Oral and Craniomaxillofacial Plastic Surgery, UKGM University Hospital Marburg, Faculty of Medicine, Philipps-University Marburg, D-35043 Marburg, Germany; 2 Department of Oral, Craniomaxillofacial and Plastic Surgery, University Hospital Ruppin-Brandenburg, Faculty of Medicine, Medical University Brandenburg, D-16816 Neuruppin, Germanyveronika.shavlokhova@gmail.com (V.S.); christian.stoll@mhb-fontane.de (C.S.); 3 Department of Microbiology, Faculty of Medicine Siriraj Hospital, Mahidol University, Bangkok 10700, Thailand; popchai@yahoo.com; 4 Chulabhorn Royal Academy, Bangkok 10210, Thailand; nithimahanonda@gmail.com; 5 Thai Association of Pediatric Dentistry, Bangkok 10400, Thailand; prim.a@chula.ac.th; 6 Royal College of Dentists of Thailand, Nonthaburi 11000, Thailand; 7 Department of Oral, Craniomaxillofacial, Aesthetic and Plastic Surgery, GLG Werner Forßmann Hospital Eberswalde—Academic Teaching Hospital of Charité Medical University Berlin, D-16225 Eberswalde, Germany; 8 Private Practice, D-73614 Schorndorf, Germany

**Keywords:** health care provider, COVID-19, vaccine booster, review

## Abstract

While the World Health Organization (WHO) has de-escalated coronavirus disease 2019 (COVID-19) from a global health emergency, ongoing discussions persist as new viral variants. This article aimed to consolidate German recommendations and international research to offer health care providers (HCPs) a comprehensive guide on COVID-19 boosters in 2024. The review outlines key recommendations from the German Robert Koch Institute. HCPs should receive COVID-19 boosters at least 12 months after their last vaccination or COVID-19 infection, contingent on the prevalent viral variant(s) in the region. However, excessive doses and/or frequent boosters, especially with mRNA vaccines, may lead to immune imprinting, T-cell exhaustion, and immunoglobulin (Ig) switching. Notably, this review highlights the significance of Ig, particularly IgA and IgG subclasses, in influencing infection risk and disease progression. Furthermore, it explores the implications of mRNA vaccine technology and potential adverse effects related to excessive dosing. In conclusion, this article provides a comprehensive analysis of COVID-19 vaccine boosters for HCPs, synthesising current recommendations, scientific debates, and considerations for optimising protection against SARS-CoV-2 in the evolving landscape of the post-pandemic era.

## 1. Introduction

The coronavirus disease 2019 (COVID-19) pandemic has posed challenges to healthcare systems globally, significantly impacting public health, economies, and daily life. Although public health measures such as maskwearing, social distancing, and lockdowns have been extensively implemented to control the virus’ spread, vaccination remains the primary prevention method. Vaccination campaigns have been used to achieve widespread immunity and mitigate the burden of COVID-19. The development of the initial vaccines brought hope for controlling the pandemic [1,2].

On 5 May 2023, the World Health Organization (WHO) no longer considered COVID-19 as a global health emergency. However, the need for ongoing vaccination efforts, including the consideration of booster doses, has been highlighted as a result of emerging variants, breakthrough infections, and evidence of waning immunity. Discussions among public health experts, policymakers, and health care providers (HCPs) have been fuelled by questions regarding the necessity, timing, and target populations for booster doses. There has been debate surrounding the necessity of boosters to enhance immune responses, prolong protection, and combat emerging variants. However, some experts advocate concerns regarding equity, vaccine distribution, and resource allocation. The changing recommendations for COVID-19 booster reflect the dynamic nature of the pandemic and the challenges of balancing public health objectives with ethical and logistical considerations [1,2].

The purpose of this article was to present a thorough overview of the current German recommendations and considerations from international research on the use of COVID-19 boosters among HCPs in 2024. The article synthesised the latest evidence and guidelines to offer insights into the rationale behind booster dose strategies, the evolving understanding of vaccine effectiveness and durability, and the implications for healthcare practice and policy. This work aimed to inform HCPs, policymakers, and the public about the latest developments in COVID-19 vaccination strategies, with a focus on optimising protection for HCPs and the communities they serve.

## 2. Current German Recommendations

On 11 January 2024, the Robert Koch Institute (RKI), responsible for managing infectious diseases in Germany, released its latest recommendations [2]. These recommendations can be summarised as follows:

1. Despite Despite the transition from a pandemic level (defined as “the infection characterised by widespread international impact causing social disruption, economic loss, and general hardship”) to the endemic level (defined as “the outbreak with the consistent presence of the disease limited to a specific region, and predictable spread and rates”), the epidemiological situation of COVID-19 remains strongly distributed around the population.

The objectives of the recommendations made by the RKI’s Standing Committee on Vaccination (STIKO) for COVID-19 are threefold: (1) to reduce the severity of symptoms, specifically targeting reductions in hospitalisation and mortality, (2) to minimise the potential long-term complications of COVID-19, and (3) to protect healthcare providers at all levels from COVID-19. 

According to the STIKO, the immunity against COVID-19 is divided into the basic and hybrid immunity (Table 1). Achieving “basic immunity” in all adults aged ≥18 years, including women of childbearing age and pregnant women without underlying diseases, requires two initial doses of vaccine and at least one booster dose. In this context, one infection is considered equivalent to one dose of vaccine. Therefore, the STIKO recommends that all adults with “basic immunity” should either receive the vaccine or be infected “at least three times” (i.e., three antigen [Ag] contacts); one of these three times should be vaccine immunity, e.g., two vaccinations plus one infection, or one vaccination plus two infections. The interval between a vaccination and an infection must be at least 3 months; otherwise the vaccination and the infection are counted as a single immunisation. 

In contrast, individuals with “hybrid immunity”, resulting from both vaccination and infection, may experience enhanced protection against severe symptoms. This phenomenon is attributed to a broader recognition of different Ags not included in the vaccine design and the subsequent boosting of mucosal immunity [2,3,4,5]. The emergence of the Omicron variant (B.1.1.529) suggests that the spike protein of this viral variant may elicit broader SARS-CoV-2 protective immunity against the emerging variant(s) [1]. Studies indicate that hybrid immunity can last for at least 12 months. Therefore, it is recommended that the next booster should not be administered before 12 months have passed since the last vaccination or infection.

2. The updated recommendations include the introduction of a new booster for specific population groups who received their last vaccination booster or had an infection 12 months ago or longer. The targeted groups are as follows:
  (1) Individuals aged ≥60 years;
  (2) Residents in nursing homes;
  (3) HCPs who have direct contact with patients and/or nursing home residents;
  (4) All individuals aged ≥6 months with underlying diseases, encompassing bedridden conditions, chronic obstructive lung disease (COPD), chronic cardiovascular, hepatic and renal diseases, diabetes mellitus and other metabolic diseases, obesity, chronic neurological diseases (e.g., dementia, stroke, mental retardation, psychological diseases), immunodeficiency (e.g., human immunodeficiency virus [HIV] infection, chronic inflammatory diseases under immunosuppressive therapy including post-organ transplantation), and active cancer;
  (5) Family members and individuals with direct contact with immunocompromised persons (e.g., after organ or stem cell transplantation, haemodialysis) aged ≥6 months.

These specific groups are identified based on increased vulnerability to severe outcomes from COVID-19 and the potential complications associated with their health conditions. The targeted approach aims to provide enhanced protection to those most at risk within the population.

3. The administration timing of the “yearly” booster is recommended during the autumn, aligning with statistical evidence indicating that disease waves tend to peak during the winter months. This strategic timing aims to maximise the effectiveness of the booster in preparation for the seasonal increase in COVID-19 cases. Additionally, it is suggested that, when indicated, the COVID-19 booster may be administered concurrently with the influenza or pneumococcal vaccine. This co-administration approach aims to optimise protection aims to optimise protection against both COVID-19 and influenza or pneumonia, particularly during periods of heightened respiratory pathogen activity, i.e., in the winter.
4. The choice of vaccine for the booster is recommended to be adapted to the prevalent viral variants in the region. The current variant in Germany is XBB.1.5 (as of 11.01.2024). In European Union (EU) countries, including Germany, the approved booster vaccines encompass various categories:
  - (monovalent) mRNA vaccines: Comirnaty Original and Comirnaty XBB.1.5 (BioNTech/Pfizer), Spikevax (Moderna; cautioned against use as a booster for individuals aged between 6 months and 5 years), and Spikevax XBB.1.5 (Moderna).

The use of monovalent vaccines during the Omicron wave has faced some hesitation. However, a randomised controlled trial conducted in the UK demonstrated that both Comirnaty Original and Spikevax were well tolerated. The fold change in anti-spike protein immunoglobulin (Ig) G titres from before (day 0: 1.59, 1.41–1.78; 2.19, 95% confidence interval [CI], 1.90–2.52) to after (day 14: 12.19, 95% CI, 10.37–14.32; 15.90, 12.92–19.58) the fourth dose was significantly greater than that 28 days after the third dose. Additionally, both mRNA vaccine groups showed significant increases in neutralising antibodies (nAb) and T-cell responses after the fourth dose (7.32, 95% CI, 3.24–16.54; 6.22, 95% CI, 3.90–9.92). The study also reported that Spikevax induced higher antibody (Ab) titres than Comirnaty Original after the fourth dose [6,7].

- bivalent mRNA vaccines: Comirnaty Original/Omicron BA.4/5 and Comirnaty Original/Omicron BA.1 (BioNTech/Pfizer), Spikevax bivalent Original/Omicron BA.4/5 and Spikevax bivalent Original/Omicron BA.1 (Moderna). Notably, Moderna vaccines should be administered carefully to individuals aged < 30 years due to the risk of peri- and myocarditis.

When comparing the monovalent Moderna with the bivalent Moderna after 28 days of the fourth dose of the bivalent vaccine as a second booster, this exhibited significantly greater nAb titres against Omicron BA.1 and other variants (BA.4/5, alpha, beta, gamma, and delta), maintaining a safety and reactogenicity profile akin to that of the monovalent vaccine [7,8].

- viral vector vaccines: Vaxzevria (AstraZeneca) and JCOVDEN (Janssen Cilag International), recommended only for individuals aged ≥60 years due to the risk of thromboembolic events in younger individuals. Moreover, JCOVDEN must not be used as a booster in individuals who previously received mRNA or viral vector vaccines.
- protein-based vaccines: Nuvaxovid (only for individuals aged ≥18 years) and Novaxovid XBB 1.5 (Novavax), seemingly suitable for young individuals due to the very low risk of peri- and myocarditis. However, there are limited scientific data on the use of these vaccines in pregnant and breastfeeding women.
- inactivated whole-virus vaccine: COVID-19 vaccine Valneva (Valneva) is recommended only for individuals with contraindications to other vaccines and unsuitable for those who have already received Valneva or viral vector vaccines as part of their basic immunisation. Limited scientific data are available regarding the use of this vaccine in pregnant and breastfeeding women.

5. Pre-exposure prophylaxis (PEP) against severe acute respiratory syndrome coronavirus 2 (SARS-CoV-2) is designed to complement vaccination and hygiene practices, not replace them. In specific cases, such as after stem cell transplantation (prior to immunological reconstitution), B-cell depletion therapies (when B-cell reconstitution is absent), CAR-T-cell therapies, severe immunosuppression (e.g., after solid organ transplantation or ongoing chemotherapy), and those with genetically-related immune defects leading to poor antiviral immunity, the use of Evusheld (AstraZeneca) may be considered. This agent comprises two monoclonal Ab, namely, tixagevimab and cilgavimab. However, it is crucial to note that the efficacy of this drug preparation is significantly reduced or non-existent against the currently circulating Omicron sublineages.
6. According to Section 22a of the German Infection Protection Law, foreign nationals residing in EU countries who have been vaccinated with vaccines not approved by the EU, such as Sinovac (Sinovac Biotech Ltd. SVA:NASDAQ GS, Beijing, China), Sinopharm (China National Pharmaceutical Group Co., Beijing, China), COVAXIN, (Bharat Biotech International Ltd., Turkapally, India), and Sputnik V (Russian Gamaleya Research Institute of Epidemiology and Microbiology), are required to repeat the EU-approved vaccination [2], even though some investigators found that the Sinopharm booster is safe and capable of rescuing immune signals and further strengthening the protective immune response by increasing nAb levels against some Omicron sublineages [7,9].

Individuals who have only received one non-EU-approved vaccine dose, including those who have received intranasal vaccines like BBV154-adenovirus-vectored vaccine (Bharat Biotech International Ltd.), are considered unimmunised and must complete the full course of EU-approved triple vaccines. For those who have received two or more non-EU-approved vaccine doses, a booster with at least one EU-approved vaccine dose is recommended. This approach aims to enhance their immunity to a level similar to individuals who have received three mRNA vaccine doses (termed as “3-dose” vaccinees). It is noteworthy that pure mRNA vaccines provide comparable protection to partial mRNA vaccines, but both offer higher protection than non-mRNA vaccines [10].

## 3. Recent Scientific Debates

Humoral and cellular immune responses following COVID-19 vaccination exhibit significant variability among individuals, influenced by factors such as age, gender, comorbidities, medication, and prior SARS-CoV-2 infections. Despite comprehensive vaccinations, certain individuals seem more susceptible to primary infections and reinfections [5], particularly concerning the Omicron variant [11].

Cellular immunity, specifically mediated by T-cells, plays a crucial role in limiting infection through viral clearance and providing clinical protection against COVID-19 [5]. In 2022, Reynolds et al. [12] introduced the concept of “hybrid immune dampening” to elucidate immune escape in individuals who received the Comirnaty Original and later contracted COVID-19. Prior imprinting from SARS-CoV-2 infection, such as exposure to ancestral or Alpha viruses, can result in significant immune dampening [4]. The concept of immune imprinting, also known as the “original antigenic sin”, refers to the immune system’s tendency to restrict its response to new variant Ag after exposure to the original Ag. Initially described for the influenza virus and later for HIV, dengue viruses, and most recently, SARS-CoV-2, this phenomenon may be relevant for both B- and T-cell immunity [5,13].

Receiving monovalent variant-based vaccines could promote broader cross-reactive Ab and overcome the limitation of immune imprinting caused by the ancestral SARS-CoV-2 spike [14]. Reynolds et al. [12] observed cross-reactive immunity in the ancestral Wuhan Hu-1 variant and two other variants of concern (VOCs), namely, B.1.1.7 (Alpha) and B.1.617.2 (Delta). This cross-reactivity was associated with the increases in Ab-binding, nAb potency, memory B-cell frequency, and T-cell recognition. Immunological imprinting from a prior Alpha infection reduced the persistence of responses to the Omicron variant, making cross-reactive boosting of immunity to Omicron less effective. However, receiving three doses of Comirnaty Original improved cross-reactive immunity to other VOCs besides Omicron. Unfortunately, infection with Omicron did not enhance immunity due to the immunological imprinting caused by a previous infection with Wuhan Hu-1 [12]. 

SARS-CoV-2 may employ evasion mechanisms to avoid immune surveillance and attack, including inhibition of interferon (IFN) synthesis, disruption of Ag presentation, evasion of Ab through nanotube construction, and induction of lymphopenia through syncytial generation [15]. Evidence indicates that changes in IFN-γ level dynamics after the booster dose are less pronounced than the humoral dynamics. However, a notable rise in IFN-γ levels was observed following the boost in infection-naïve individuals and those infected with the Omicron variant, but not in those infected prior to the Omicron wave [5]. 

These findings suggest that the immune system may be significantly subverted and differentially modulated through immunological imprinting, leading to what is referred to as “hybrid immune dampening”. This may help explain why individuals who received three doses of the Comirnaty Original vaccine experienced breakthrough infections and frequent reinfections during the Omicron wave [4,16]. Caution is advised when interpreting the results of this study, especially in cases involving other vaccines. 

Another hypothesis suggests that higher Omicron-specific immunity after the third mRNA vaccine was observed in those with prior SARS-CoV-2 infection. This may lead to lower viral loads and reduced antigenic exposure during the Omicron infection, resulting in muted immune induction [4]. In a study conducted in Denmark with a large sample size (*n* = 1325), almost half of the individuals who had not been previously infected with Omicron contracted the virus for the first time after receiving the booster dose [5]. 

However, a recent study by a Chinese research group analysed the impact of sequential reinfections with the Omicron variant on nAb profiles (*n* = 93). The authors found that breakthrough infections caused by the Omicron variant, particularly reinfections by different subvariants, were more effective in inducing neutralisation against new variants. The neutralisation geometric mean titres of nAb for the ancestral variant and four other variants (BA.2.2, BA.5.2, BF.7, and XBB.1) differed greatly between breakthrough infections and homologous booster subjects. Reinfection elicited the highest Ab levels for all other four variants, but with a relatively lower titre for the Wuhan strain. Repeated stimulation by the Omicron variant may significantly enhance the magnitude and breadth of neutralisation activity against it. This could result in broader nAb profiles and a reduction in the duration of viral shredding. These effects may overcome the adverse effects of immune imprinting induced by previous monovalent vaccination [1].

The concept of “T-cell exhaustion” related to reduced immunity in cases of very high doses and/or too frequent administration of COVID-19 vaccines, particularly mRNA types, has been proposed [16,17]. T-cells play an essential role in protection against severe COVID-19, and repeated exposure to SAR-CoV-2-Ag delivered by vaccination may lead to T-cell exhaustion or dysfunctionality [18]. A small case series from Thailand (*n* = 90) suggested that T-cell exhaustion in individuals with exaggerated vaccinations can impede T-cell functions after booster vaccinations, especially after a short interval of revaccination (i.e., 1–3 months), and may cause herpes zoster reactivation due to transient lymphopenia [17]. This evidence supports STIKO’s recommendations that the new booster in 2024 should be administered 12 months (or longer) after the last vaccination or infection [2], particularly in individuals with “hybrid immunity” characterised by long-term memory against SARS-CoV-2 [18]. However, COVID-19 patients may also experience exhaustion of immune cells, such as T-effector cells (Teff), T-regulatory cells (Tregs), natural killer cells (NKs), and Ag-presenting cells (APCs), due to the upregulation of inhibitory immune checkpoints (ICs). Clinical studies have shown a significant relationship between T-cell exhaustion and the expression of ICs, which can accelerate COVID-19 virulence [19]. Therefore, dysfunctional T-cell immunity, regardless of its cause, not only produces immune waning after the booster vaccination, but also increases the virulence of the disease if an individual with “T-cell exhaustion” is infected again by SARS-CoV-2.

The interplay between Ig and COVID-19 remains a subject of ongoing debate. Among the five classes of Ig found in humans—namely, IgM, IgD, IgA, IgE, and IgG—humoral responses have proven more impactful for viral transmission and breakthrough infections than cellular responses [5]. In particular, IgA assumes a pivotal role in facilitating viral clearance during breakthrough SARS-CoV-2 infections [14]. 

At the mucosal barrier, secretory IgA (sIgA) serves as the primary line of defence against invading pathogens. Its functions include preventing pathogen entry, binding and excreting pathogens across epithelial lumens, reducing viral load, and modulating inflammation in subepithelial tissues. While an IgA response is triggered following SARS-CoV-2 infection and intramuscular vaccination, there is a notable inadequacy in generating long-lasting mucosal Ab responses, even among individuals who have recovered from the disease. This deficiency, especially in mucosal nAb and IgA responses, likely contributes to the high incidence of breakthrough infections with the Omicron variant, underscoring the critical need for effective mucosal COVID-19 vaccines [14]. 

In contrast, IgG stands out as the most abundant protein in human serum, constituting 10–20% of plasma protein. It is divided into four subclasses—IgG1, IgG2, IgG3, and IgG4—each characterised by unique profiles concerning Ag binding, immune complex formation, complement activation, triggering of Teff, half-life, and placental transport [20]. Real-world data support a significant correlation between increased IgG and nAb titres and reduced infection probability and progression to symptomatic disease with Omicron and its sublineages. Notably, each 10-fold increase in IgG titre is associated with nearly 30% lower odds of infection and each 2-fold increase in nAb titres correlates with an 11% reduction in odds of infection with Omicron and its sublineages. Additionally, the odds of substantial symptomatic disease are reduced for each 10-fold increase in IgG levels and for each 2-fold increase in nAb levels [20].

Research has shown that Ig titres typically decrease six months after receiving the mRNA booster dose [16,21]. Low levels of IgG, IgA, and nAb, but not T-cell responses, increase the risk of future SARS-CoV-2 infections [5]. Injecting multiple doses of mRNA vaccine shots may result in the production of IgG4 instead of IgG1 and IgG3, as well as impaired activation of CD4^+^ and CD8^+^ T-cells. Furthermore, individuals who have received three doses of Comirnaty Original may experience a reduction in Ab-mediated phagocytic activity and complement deposition. This could potentially increase the risk of other opportunistic infections [15,22,23,24].

One unique characteristic of IgG4 is its ability to undergo a “Fab arm exchange” process. This process involves swapping one of the Ag-binding regions (Fab arms) with another IgG4 molecule, resulting in the formation of Ab with two different specificities. The Fab arm exchange can reduce the overall antiviral efficacy of IgG4 antibodies, including viral clearance, compared to IgG1 and IgG3. Elevated ratios of IgG4 to IgG1 are associated with unfavourable disease outcomes, potentially leading to prolonged viral persistence and increased susceptibility to the spike protein of SARS-CoV-2 [15,22,23,24].

Reinfection with SARS-CoV-2 may also weaken the immune response due to IgG4-induced immune tolerance. Furthermore, the virus can cause chronic infection by infecting cells for an extended period [23], which may be influenced by consecutive events of “Ig class switch recombination” and the maturation of IgG4-switched memory B-cells. It is important to note that this phenomenon is not observed when using the viral vector-based Vaxzevria [22]. 

Moreover, an elevated concentration of serum IgG4 and the presence of IgG4-positive plasma cells in tissues may be pathological and linked to various IgG4-related diseases, including autoimmune hypophysitis, Riedel’s thyroiditis, interstitial pneumonitis, inflammatory aortic aneurysm, retroperitoneal fibrosis, type 1 autoimmune pancreatitis, Mikulicz’s disease, idiopathic tubulointerstitial nephritis, prostatitis, and orbital, pulmonary and paratesticular inflammatory pseudotumour [20,21,23]. 

On one side, the use of mRNA technology offers many advantages over conventional vaccine and drug development, including short manufacturing time and diversity of applications through simple changes in mRNA sequence [25]. On the other hand, administering excessive doses and/or frequent boosters of mRNA vaccines may reduce immune protection and trigger various pathological conditions. It is crucial to acknowledge that IgG4 may not be the sole pathway for SARS-CoV-2 clearance. Other innate immune mechanisms stimulated by hybrid and herd immunity might prove more effective against SARS-CoV-2 [15,22,23,24]. 

Replacing uracil with N1-methyl pseudouridine in the genetic code may reduce cellular immunity by activating regulatory T-cells through the modified protein. Additionally, the spike protein on exosomes can persist for more than four months. The mRNA-encapsulating lipid nanoparticles (LNPs) can cause inflammation and accumulate in the liver, spleen, adrenal gland, and ovaries. These LNPs contain many buffer and small-molecule lipid components that are also capable of inducing toxicities. Moreover, vaccination can significantly disrupt IFN-1 signalling, resulting in adverse effects on human health. When immune cells take up the vaccine LNPs, they release exosomes into circulation, carrying the spike protein and critical microRNAs. These exosomes trigger signalling responses in recipient cells at distant locations. The findings suggest disruptions in the regulatory control of protein synthesis and cancer surveillance, which may be related to various health conditions such as neurodegenerative diseases, myocarditis, immune thrombocytopenia, Bell’s palsy, liver disease, impaired adaptive immunity, compromised DNA damage response, and the initiation of tumourigenesis [24,25]. 

## 4. Conclusions

The intricacy of immune responses to SARS-CoV-2 has grown with the introduction of various vaccine boosters, multiple Ag exposures, and frequent virus mutations. This complexity poses challenges for future vaccination campaigns. It is imperative to evaluate both humoral and cellular responses and establish a consensus on the correlates of protection. This is crucial for identifying individuals eligible or suitable for additional vaccine boosters [5]. 

While debates persist regarding the necessity of further boosters, HCPs face an elevated risk of infection. COVID-19 boosters in these individuals aim to reduce their occupational risk of infection and transmission to vulnerable populations. Bivalent boosters may provide protection against hospitalisation and severe symptoms caused by various SARS-CoV-2 variants [7,8,26,27,28], particularly in individuals with “hybrid immunity” [1,2,3,4,5,7,8]. Such boosters can enhance both humoral and cellular immunity, decrease infection rates, and offer protection, especially for specific populations such as organ transplant recipients, dialysis patients, individuals with chronic renal disease, the elderly population, and immunocompromised individuals [7]. 

However, the type, dosage, and timing of boosters should be personalised for each individual. Regional and national organisations, such as medical and dental councils, should continue researching repeated seasonal boosting while actively promoting booster vaccinations. 

## Figures and Tables

**Table 1:** The immunity types according to the German Standing Committee on Vaccination (STIKO) [2].

Basic Immunity	Hybrid Immunity
- 2 initial doses + 1 booster dose	- 2 initial doses + 1 infection - 1 initial dose + 1 infection + 1 booster dose - 1 initial dose + 2 infections - 1 infection + 1 initial dose + 1 booster dose - 1 infection + 1 initial dose + 1 infection - 2 or 3 infections + 1 booster dose

Note: (1) It is mandatory that there be a minimum of three months between each event. (2) This classification is applicable to individuals who have received mRNA vaccines or the viral vector AstraZeneca vaccine based on merit. (3) Individuals who have had COVID-19 three times previously require a booster dose to achieve hybrid immunity [3].

## Data Availability

The data supporting the findings of this study are available upon reasonable request from the corresponding author.
